# Tiredness after work associates with less leisure-time physical activity

**DOI:** 10.1038/s41598-024-58775-4

**Published:** 2024-04-04

**Authors:** Tanja Sjöros, Jooa Norha, Riitta Johansson, Saara Laine, Taru Garthwaite, Henri Vähä-Ypyä, Eliisa Löyttyniemi, Kari K. Kalliokoski, Harri Sievänen, Tommi Vasankari, Juhani Knuuti, Ilkka H. A. Heinonen

**Affiliations:** 1 Turku PET Centre, University of Turku and Turku University Hospital, P.O. Box 52, 20521 Turku, Finland; 2 The UKK Institute for Health Promotion Research, Tampere, Finland; 3 Department of Biostatistics, University of Turku and Turku University Hospital, Turku, Finland; 4 Faculty of Medicine and Health Technology, Tampere University, Tampere, Finland

**Keywords:** Risk factors, Occupational health, Public health

## Abstract

Physical activities and sedentary behaviors take place in different contexts. This study aimed to determine if the context, total score, and leisure-time MET-index assessed by the Baecke questionnaire associate with each other or with sedentary behavior and physical activity outcomes from a 4-week accelerometer measurement in physically inactive adults with overweight. The item *“After working I am tired”* correlated negatively with items related to leisure-time physical activity and sports participation. The total Baecke Score showed weak but significant correlations with accelerometer-measured sedentary behavior, physical activity, daily steps, and mean activity intensity of the day (r = − 0.33, 0.41, 0.35, and 0.41, respectively). The associations strengthened when the Sport Index was omitted from the Score. The leisure-time MET-Index did not correlate with accelerometer-measured sedentary behavior or physical activity. Tiredness after working associated with less self-reported physical activity during leisure time. This suggests that better recovery from work-related stress could increase leisure-time physical activity, or increasing leisure-time physical activity could reduce tiredness after working. Moreover, among self-reportedly inactive adults with overweight, focusing the questionnaire on work and non-sport leisure time instead of total time might give more accurate estimates of sedentary behavior and physical activity when compared to accelerometry.

The study is registered at ClinicalTrials.gov (NCT03101228, 05/04/2017).

## Introduction

Sedentary behavior (SB) is defined as a sitting, lying, or reclining posture combined with a low energy expenditure^1^. SB is associated with several health problems as well as mortality^2–4^. SB is also associated with poorer mental well-being, as well as with symptoms of depression and anxiety, among people that are physically inactive^5,6^. Moreover, physical inactivity is a growing health concern worldwide^7^. Device-based estimates of SB, preferably derived from accelerometry and/or inclinometry, are considered more valid and reliable compared to questionnaire-based estimates, which may be affected by reporting bias^8^. The best accuracy is likely achieved by using both device and questionnaire-based estimates^9^. However, globally, the accessibility of accelerometers is limited, and therefore inexpensive and easily implementable methods, such as questionnaires, are needed to reliably assess both SB and physical activity (PA)*.*

The Baecke habitual physical activity questionnaire is frequently used although it dates back several decades^10–14^. The questionnaire offers numerous advantages; it assesses multiple PA contexts, such as work, leisure, and sports participation. Indexes for these three behaviors can be calculated and combined into a total Baecke Score, while individual items can also be evaluated separately. Compared to accelerometers, the Baecke questionnaire also has the advantage of providing information on subjective feelings, such as tiredness, which may play a role in PA and SB. Indeed, previous studies show that vigorous PA may be followed by prolonged SB and high job strain associates with less leisure-time PA^15,16^. Therefore, feeling tired after working may associate with less PA. However, it is not known whether the contexts in which PA is accumulated (i.e., work or leisure) assessed with the Baecke questionnaire correlate with each other.

The test–retest reliability of questionnaire-based estimates of SB and PA is generally acceptable (median intraclass correlation coefficient 0.77 in adults), but the validity against device-measured estimates remains modest (median correlation 0.27–0.28 in adults)^17^. However, the Baecke questionnaire has performed relatively well in comparison to many other frequently used questionnaires (r = 0.69 for correlation between the Baecke score and doubly-labelled water-measured total energy expenditure)^17^. The total Baecke score correlated positively with accelerometer-measured PA (r = 0.47), measured with triaxial accelerometers over four days among 40-year old men^18^; and over five days among adults with medium or high education level (r = 0.34 and 0.38, respectively)^11^. Additionally, the correlation between the Baecke Score and PA level measured with doubly labelled water was even higher (r = 0.69)^19^. However, in the studies above, the accelerometer assessment of PA has lasted only 4–5 days and the association with accelerometer-measured SB remains unexamined.

Therefore, the purpose of this study was to assess how the Baecke score, indexes, or individual items associate with accelerometer-measured PA and SB in self-reportedly physically inactive (not meeting PA guidelines^20^) adults. Additionally, to investigate the interrelations between PAs and SBs in different contexts, we assessed the correlations between the individual Baecke items. Moreover, we aimed to determine which Baecke items have the best predictive value for accelerometer-measured SB. Finally, differences in questionnaire-based PA estimates between men and women were studied.

## Methods

This study was a one-arm observational study consisting of the screening phase of an intervention study that is registered at ClinicalTrials.gov (NCT03101228, 05/04/2017). The study was conducted at the Turku PET Centre, Turku, Finland between April 2017 and May 2019. This study was conducted according to good clinical practice and the Declaration of Helsinki. The participants gave their informed consent before entering the study. The study was approved by the Ethics Committee of the Hospital District of Southwestern Finland (16/1801/2017).

### Participants

The participants in this study were recruited from the local community by newspaper advertisements and bulletin leaflets as previously described^21^. The inclusion criteria were: Age 40–65 years, BMI 25–40 kg/m^2^ and, according to self-reports during a screening interview, not meeting the current recommendations for PA (< 120 min/week of moderate-to-vigorous PA) and sitting most of the day. The exclusion criteria were: history of a cardiac event, diagnosed diabetes, abundant use of alcohol (according to national guidelines), use of narcotics, smoking of tobacco or consumption of snuff tobacco, inability to understand written Finnish and any chronic disease or condition that could create a hazard to the subject safety or endanger the study procedures.

### The Baecke habitual physical activity questionnaire

The Baecke Habitual Physical Activity Questionnaire has 16 individual items, all of which are ranked on a five-point scale^10^. The total sum score of the questionnaire (Baecke Score, 3–15) as well as Work, Leisure, and Sport Indexes (1–5) were calculated based on the individual items^10^. Additionally, as the Sport Index correlated poorly with the accelerometry, the Work and Leisure Indexes were combined to further elucidate the correlations. Because of missing data and inaccurate reporting (e.g., reporting profession/education instead of the actual occupation), the first item (occupation) was left out from the Work Index. Moreover, interindividual differences in PA within a given occupation was already identified as a potential bias in the original publication by Baecke and colleagues^10^. As a result, in the current study, the Work Index (1–5) contained 7 items, and both the Sport Index and Leisure Index (1–5) contained 4 items. For the calculation of simple sport score (item 9), the intensities of different sports were classified on a 3-point scale (0.76, 1.26, 1.76 MJ/h), as originally described^10^. The reported sport activities were classified with the aid of the original publication’s examples and the Compendium of Physical Activities^22^. The most frequently reported sports activity was walking, which was classified as a light intensity sport (0.76 MJ/h)^22^. Additionally, for example yoga and pilates were classified as light intensity sports, Nordic walking, strength training, and group exercise were classified as medium intensity sports (1.26  MJ/h), and crossfit and ice hockey as high intensity sports (1.76 MJ/h)^22^.

### The MET-index

A leisure-time MET-index was calculated from additional questions as previously described^23,24^. In short, the participants were asked about their leisure-time physical activity intensity, duration, and frequency. The reported intensity was converted to metabolic equivalents (MET), and a product of activity intensity × duration × frequency was calculated and presented as leisure-time MET h/day (MET-Index).

### Accelerometry

SB and PA were measured for four weeks with hip-worn tri-axial accelerometers (UKK AM30, UKK Terveyspalvelut Oy, Tampere, Finland) as previously reported^21^. Briefly, the collected accelerometer data was analyzed in six-second epochs using the validated mean amplitude deviation (MAD) method^25,26^. SB and standing were defined based on the MAD as < 1.5 MET (MAD < 22.5 m*g*), light PA was defined as ≥ 1.5–3.0 MET (MAD 22.5–91.5 m*g*), and moderate to vigorous PA as ≥ 3.0 MET (MAD > 91.5 m*g*). Additionally, the daily mean MET mean value was calculated.

The body posture was determined with angle for posture estimation (APE) method only for the 6-s epochs with < 1.5 MET^27^. The Earth’s gravity vector detected during walking was used as the reference vector (i.e., zero degrees) for the APE algorithm. The epochs with an APE value < 11.6° (as compared to the reference vector) were classified as standing and epochs having APE values ≥ 11.6° were classified as SB. In free-living conditions, the agreement between the posture classification from simultaneous thigh-worn and hip-worn data has been about 90%^27^.

The participants were advised to wear the device only during waking hours. Wear time of 10–19 h/day and 4 days of measurement were considered valid. Measurement time exceeding 19 h/day likely means that the accelerometer was worn during sleeping and thus, time exceeding 19 h/day was removed from SB. Additionally, proportions of SB and different activity intensities per day (percentage of daily accelerometer wear time) were calculated for the correlation analyses to adjust for the confounding effect of variation in the wear time.

### Statistical methods

The associations between accelerometer-measured SB and PA and self-assessed PA with the Baecke questionnaire Score and Indexes were examined with Pearson’s correlation coefficient. The normal distributions of the variables were assessed visually and with Shapiro–Wilk’s test. Logarithmic transformations (log10) were made when necessary to fulfil the normal distribution assumption of the variables. The associations between individual questionnaire items were examined with Spearman’s rank correlation coefficient. The coefficient of determination (R^2^) of the individual items with strongest correlation to accelerometer-measured SB was further examined using linear regression analysis. The regression model was created stepwise entering all the items that had a significant correlation with accelerometer-measured SB (items 1, 2, 3, 6, 17, and 18) and stepwise removing non-significant items from the model. The final model included the items 2, 3, and 18, of which item 3 was not significant (p = 0.11), but was included in the final model because it slightly improved the coefficient of determination and walking at work would theoretically associate with less SB. Variance inflation factors < 5 were considered as no significant multicollinearity.

The differences between men and women were examined with independent samples t-test or Mann–Whitney U test when applicable. If not otherwise stated, data are expressed as means (SD). The level of statistical significance was set at 5% (two-tailed). All analyses were carried out with the IBM SPSS Statistics versions 26.0–28.0.

### Ethical approval

The study was approved by the Ethics Committee of the Hospital District of Southwest Finland (16/1801/2017).

### Informed consent

The participants gave their informed consent before entering the study.

## Results

The participant characteristics are presented in Table 1. The more detailed metabolic characteristics of the participants have been previously published^21^. The Pearson correlation coefficients of The Baecke Score and Work, Leisure, and Sport Indexes as well as the MET-index and accelerometer-measured SB and PA are presented in Table 2. The Baecke Score correlated negatively with accelerometer-measured SB (r = − 0.33, p < 0.001), and positively with LPA, MVPA, total PA, daily steps, and MET mean (r = 0.36, 0.34, 0.41, 0.35, and 0.41, respectively, p < 0.001 for all). The Work Index had the strongest correlations to LPA and total PA (r = 0.45 and 0.38, respectively, p < 0.001). The Leisure Index had the strongest correlations to daily steps and MET mean, (r = 0.34, p < 0.001 in both outcomes). The sum of Work and Leisure Indexes had the strongest correlations to accelerometer-measured total PA, LPA, MET mean, and SB (r = 0.46, 0.43, 0.42, and − 0.40, respectively, p < 0.001 for all). The leisure-time MET-index (calculated from additional questions) was not significantly associated with accelerometer-measured SB or PA (Table 2).

**Table 1:** Study participant characteristics. If not otherwise stated, the results are reported as mean (SD).

	All	Women	Men	p
n (% of total)	144 (100)	102 (71)	42 (29)	
Age, years	56.9 (6.5)	56.4 (6.7)	58.0 (6.0)	0.16
Height, cm	169.2 (8.9)	165.2 (6.1)***	178.8 (7.1)***	< 0.001
Body mass, kg	91.1 (15.4)	86.7 (13.4)***	101.8 (14.7)***	< 0.001
BMI, kg/m^2^	31.7 (4.0)	31.7 (4.2)	31.8 (3.6)	0.91
Accelerometry, days	25 (4)	26 (4)	24 (5)	0.16
Wear time, h/day	14.37 (1.04)	14.41 (1.00)	14.27 (1.14)	0.45
Sedentary time, h/day	9.62 (1.32)	9.42 (1.31)**	10.13 (1.24)**	0.003
Standing, h/day	1.97 (0.76)	2.18 (0.76)***	1.44 (0.44)***	< 0.001
LPA, h/day	1.78 (0.51)	1.83 (0.45)	1.67 (0.61)	0.082
MVPA, h/day	1.00 (0.38)	0.98 (0.36)	1.03 (0.43)	0.46
PA, h/day	2.78 (0.77)	2.81 (0.70)	2.70 (0.92)	0.44
Steps, n/day	5265 (2113)	5206 (2046)	5408 (2288)	0.61
MET mean/day	1.3 (0.1)	1.3 (0.1)	1.3 (0.1)	0.73
Baecke Score 3–15	7.41 (1.10)	7.40 (1.08)	7.43 (1.17)	0.87
Work index 1–5	2.47 (0.55)	2.51 (0.53)	2.37 (0.60)	0.12
Leisure index 1–5	2.72 (0.62)	2.70 (0.64)	2.76 (0.59)	0.49
Sport index 1–5	2.22 (0.49)	2.19 (0.52)	2.30 (0.41)	0.11
Leisure MET index, MET h/day^#^	0.60 (0.20, 1.15)	0.60 (0.16, 1.00)	0.50 (0.20, 1.4)	0.55

The differences between men and women were tested with independent samples t-test.

*BMI* body mass index, *LPA* light physical activity, *MVPA* moderate to vigorous physical activity, *PA* physical activity (LPA & MVPA combined), *MET* metabolic equivalent.

***p < 0.001; **p < 0.01.

^#^Presented as median (Q1, Q3) and gender difference tested with log10-transformed estimates.

**Table 2:** Pearson correlation coefficients between accelerometer-measured sedentary behaviour and physical activity and Baecke Score, Work, Leisure, and Sport Indexes as well as a leisure-time MET-index.

	SB %	Standing %	LPA % #	MVPA %	PA % #	Steps	MET mean	MET-index #	Baecke Score	Work Index #	Leisure Index	Sport Index	Work & Leisure Sum
SB %	1	− 0.81**	− 0.72**	− 0.66**	− 0.82**	− 0.65**	− 0.74**	0.04	− 0.33**	− 0.34**	− 0.26**	− 0.01	− 0.40**
Standing %		1	0.30**	0.28**	0.35**	0.32**	0.31**	− 0.12	0.10	0.14	0.12	− 0.09	0.16
LPA % #			1	0.41**	0.87**	0.37**	0.60**	0.04	0.36**	0.45**	0.20*	0.03	0.43**
MVPA %				1	0.79**	0.95**	0.96**	0.06	0.34**	0.20**	0.30**	0.16	0.35**
PA % #					1	0.74**	0.89**	0.06	0.41**	0.38**	0.30**	0.11	0.46**
Steps						1	0.93**	0.07	0.35**	0.14	0.34**	0.18*	0.35**
MET mean							1	0.09	0.41**	0.27**	0.34**	0.17*	0.42**
MET-index #								1	0.26**	− 0.13	0.18*	0.52**	0.04
Baecke score									1	0.59**	0.74**	0.67**	0.91**
Work index #										1	0.08	0.11	0.69**
Leisure index											1	0.33**	0.77**
Sport index												1	0.29**
Work & leisure sum													1

*SB* sedentary behavior measured by accelerometry, *LPA* light physical activity measured by accelerometry, *MVPA* moderate to vigorous physical activity measured by accelerometry, *PA* physical activity (LPA + MVPA) measured by accelerometry, *MET* mean mean metabolic equivalent (MET) of the day measured by accelerometry, *MET*-index leisure-time MET h/day (activity intensity × duration × frequency) measured by a questionnaire, *Baecke score* sum score measured by Baecke Habitual Physical Activity Questionnaire, *Work Index* working score measured by Baecke Habitual Physical Activity Questionnaire, *Leisure Index* leisure-time score measured by Baecke Habitual Physical Activity Questionnaire, *Sport Index* sport score measured by Baecke Habitual Physical Activity Questionnaire, *Work & Leisure sum* sum of Work and Leisure Indexes measured by Baecke Habitual Physical Activity Questionnaire.

^#^Analysed with log10-transformed estimates.

*Significant at the level of p < 0.05.

**Significant at the level of p < 0.01.

The correlation coefficients (Spearman’s rho) of the individual items of the Baecke questionnaire with accelerometer-measured SB and PA are presented in Table 3. Of the individual Items, the strongest predictors of accelerometer-measured SB were Items “*At work I stand*”, “*At work I walk*”, and “*During leisure time I cycle*”, together explaining about 18% (i.e., R^2^ = 0.18) of the accelerometer-measured relative SB (Table 4). Even though “*At work I walk*” was not a statistically significant item in the model, it improved the regression correlation coefficient and thus was included in the final model. Item 6, “*After working I am tired*” did not correlate with accelerometer-measured SB or PA. However, it had significant negative correlations with items considering leisure-time PA (active commuting, cycling, and walking) and sports (playing sports and PA compared to peers).

**Table 3:** Spearman’s rank correlation coefficients between accelerometer-measured sedentary behavior and physical activity and individual items of the Baecke habitual physical activity questionnaire.

	SB %	Standing %	LPA %	MVPA %	PA %	Steps	MET mean	B 2	B 3	B 4	B 5	B 6	B 7	B 8	B 9	B 10	B 11	B 12	B 13	B 14	B 15	B 16
SB %	1	− 0.80**	− 0.66**	− 0.66**	− 0.79**	− 0.66**	− 0.74**	0.17*	− 0.29**	− 0.30**	− 0.10	− 0.10	− 0.21*	− 0.17*	0.05	− 0.04	− 0.04	− 0.02	0.13	− 0.17*	− 0.24**	− 0.15
Standing %		1	0.26**	0.28**	0.32**	0.33**	0.30**	− 0.07	0.19*	0.14	− 0.09	0.16	− 0.03	0.01	− 0.13	− 0.08	− 0.08	− 0.09	− 0.05	0.08	0.15	0.07
LPA %			1	0.42**	0.84**	0.36**	0.59**	− 0.29**	0.29**	0.35**	0.26**	0.09	0.40**	0.29**	− 0.08	0.09	0.00	0.01	− 0.09	0.02	0.20*	0.12
MVPA %				1	0.81**	0.94**	0.96**	− 0.10	0.15	0.26**	0.09	− 0.03	0.13	0.17*	0.14	0.10	0.09	0.09	− 0.14	0.23**	0.21*	0.16
PA %					1	0.75**	0.92**	− 0.22**	0.28**	0.35**	0.20*	0.02	0.30**	0.25**	0.01	0.10	0.06	0.07	− 0.16	0.16	0.25**	0.19*
Steps						1	0.92**	− 0.06	0.15	0.24**	0.07	− 0.06	0.10	0.13	0.17*	0.13	0.07	0.14	− 0.09	0.26**	0.24**	0.20*
MET mean							1	− 0.15	0.21*	0.30**	0.12	− 0.03	0.20*	0.19*	0.11	0.14	0.10	0.11	− 0.16	0.24**	0.24**	0.22**
B 2								1	− 0.46**	− 0.34**	− 0.29**	0.04	− 0.39**	− 0.35**	− 0.03	− 0.01	0.01	0.03	− 0.03	0.01	− 0.17*	− 0.12
B 3									1	0.523**	0.272**	0.08	0.262**	0.327**	0.11	− 0.01	0.06	0.05	− 0.10	0.04	0.11	0.05
B 4										1	0.27**	0.09	0.29**	0.35**	− 0.01	0.02	0.12	0.17*	− 0.02	0.12	0.22**	0.11
B 5											1	− 0.03	0.55**	0.46**	0.07	0.12	− 0.07	0.10	0.08	-0.06	0.09	0.03
B 6												1	0.12	− 0.07	− 0.17*	− 0.22**	− 0.05	− 0.13	− 0.01	− 0.17*	− 0.20*	− 0.17*
B 7													1	0.44**	− 0.01	0.13	0.18*	0.10	0.08	0.06	0.06	0.10
B 8														1	0.05	0.22*	− 0.02	0.15	0.10	0.06	0.08	0.02
B 9															1	0.30**	0.15	0.47**	0.00	0.24**	0.15	0.07
B 10																1	0.19*	0.41**	0.07	0.26**	0.19*	0.23**
B 11																	1	0.28**	− 0.23**	0.27**	0.15	0.15
B 12																		1	− 0.03	0.41**	0.09	0.12
B13																			1	− 0.11	− 0.02	− 0.03
B 14																				1	0.19*	0.33**
B 15																					1	0.24**
B 16																						1

*SB* sedentary behavior measured by accelerometry, L*PA* light physical activity measured by accelerometry, *MVPA* moderate to vigorous physical activity measured by accelerometry, *PA* physical activity (LPA + MVPA) measured by accelerometry, *MET* metabolic equivalent (MET) of the day measured by accelerometry, *B2-B16* individual Items of the Baecke questionnaire, *B2* sitting at work, *B3* standing at work, *B4* walking at work, *B5* lifting heavy loads at work, *B6* being tired after work, *B7* sweating at work, *B8* physical heaviness of work, *B9* simple sport score, *B10* physical activity during leisure, *B11* sweating during leisure, *B12* playing sport during leisure, *B13* watching TV during leisure, *B14* walking during leisure, *B15* cycling during leisure, *B16* cycling/walking to school/work/shopping.

*Significant at the level of p < 0.05.

**Significant at the level of p < 0.01.

**Table 4:** Linear regression results of the individual questionnaire Items predicting the amount of accelerometer-measured sedentary behavior.

	B	95% CI	β	p-value	VIF
At work I stand	− 0.023	− 0.042, − 0.004	− 0.234	0.016	1.5
At work I walk	− 0.016	− 0.037, 0.004	− 0.158	0.110	1.6
During leisure time I cycle	− 0.017	− 0.032, − 0.003	− 0.188	0.021	1.1
R^2^ = 0.181, Adjusted R^2^ = 0.163					

*B* regression coefficient, *CI* confidence interval, *β* standardized regression coefficient, *VIF* variance inflation factor, *R*^*2*^ coefficient of determination.

We also tested whether there were gender differences in the measured outcomes. Men had more accelerometer-measured SB and women had more standing time (Table 1), as previously reported^21^. There were no significant gender differences in the Baecke Score or Work, Leisure, and Sport Indexes (Table 1). Out of individual items, there was a significant difference between men and women only in the item 10, “*In comparison with others of my age, my physical activity during leisure is more/less*”, with men reporting being more physically active than women (Mann–Whitney U, p = 0.006). However, the majority of both men (57%) and women (52%) of the current study estimated being less physically active compared to others of the same age (rank 2), but only 5% of men and 25% of women estimated being much less active (rank 1). Moreover, 38% of men estimated being equally active compared to others, but only 20% of the responded women estimated similarly (rank 3). No responders estimated being much more active compared to others (rank 5), and no men and only 3% of women estimated being more active (rank 4).

## Discussion

In this study, we showed that the Baecke Habitual Physical Activity Questionnaire total score correlates with accelerometer-measured SB and PA in a self-reportedly physically inactive population. Moreover, the correlations were stronger when considering only the sum of Work and Leisure indexes without the Sports index. Out of the individual Baecke items feeling tired after work associated with less leisure-time PA.

Compared to an earlier study in a population-based sample of 40-year-old men, the correlations of The Baecke Score with accelerometry-derived estimates of PA were slightly weaker in the current study^18^. This may be explained by different study populations; the participants of the current study were self-reportedly inactive (not meeting the current guidelines for PA), therefore the study sample was more homogenous, probably leading to less variation in self-assessed and accelerometer-measured PA and thus to weaker correlations. On the other hand, the duration of accelerometer data collection was markedly longer in the current study compared to the previous study. Moreover, the participants in the current study were older (mean age 40 vs. 57 years). However, compared to a more recent study among a sample of Brazilian adults with medium or high education level, the correlation between the Baecke score and accelerometer-measured PA (r = 0.34 and 0.38, respectively) was slightly stronger in the current study^11^.

The item “*After working I am tired*” did not correlate with any of the accelerometer-derived outcomes. A large proportion of the participants (n = 53) reported being sometimes tired after working. The item probably reflects physical, mental, and social strenuousness of the work, of which the latter two cannot be estimated with accelerometers, and therefore no significant correlations were observed. Leaving this item out of the Work Index slightly strengthened the associations with accelerometer-measured total PA, MVPA, step count, and MET mean (data not shown), but the associations with SB and LPA remained similar. Interestingly, there was a weak negative correlation between this item and the items 9, 10, 14, 15, and 16, indicating that the individuals who felt more tired after working had less sports participation and leisure-time PA. In fact, vigorous PA is often followed by increased SB which may reflect increased tiredness and less PA after a physically strenuous workday^16^. This may be of importance, because it suggests, that better recovery from work-related stress might increase leisure-time PA. Alternatively, increasing leisure-time PA could reduce tiredness after working. Both of these presumptions are supported by a finding, that a change in health behavior predicts future subjective well-being and vice versa in a 9-year follow-up^28^. Additionally, PA interventions have been able to increase work ability^29^.

Out of individual items, the strongest predictors of accelerometer-measured SB were the items “*At work I stand*”, “*At work I walk*”, and “*During leisure time I cycle*”. However, together they could explain only about 18% of the variation in accelerometer-measured SB. The item “*At work I sit*” correlated with accelerometer-measured SB very weakly (rho = 0.17). Thus it seems that no individual items of the Baecke questionnaire should be used as indicators of daily SB. In the current study, the sum of Work and Leisure Indexes was the best indicator of accelerometer-measured SB (r = − 0.40). This is in agreement with the finding that single-item questionnaires performed more poorly than multi-item questionnaires when compared to device-measured estimates of SB^8^. However, the Baecke Score performed only moderately in comparison to accelerometer-measured SB in the current study (r = − 0.33). Therefore, the Baecke Score seems to be more useful in estimating daily PA (Baecke Score vs. total PA r = 0.41), as it was originally designed for. However, parts of the questionnaire may also be useful in estimating daily SB to some extent, but they should be interpreted with caution.

One issue to consider is the absence of screen time estimation during leisure time in the original Baecke questionnaire. During the time of the release of the questionnaire, at the beginning of the 1980’s, this was hardly an issue. In the modern era, other screen behaviors have at least partly replaced TV-viewing time, especially in the younger age groups. However, the participants of the current study were adults and older adults (age 40–65), and TV still plays a major role in their leisure time, because 77% of the participants reported watching TV often or very often.

Despite its age, the Baecke questionnaire is still being frequently used in scientific research. For example, the Baecke Score and Work, Leisure, and Sport Indexes have recently been used to evaluate the genetic associations of habitual PA and health in youth^12^ and in young and older adults^14^. Questionnaires have the advantage that PA and SB can be classified into work and leisure, whereas using solely accelerometry, information about the context of PA and SB remains unknown.

In comparison with the Baecke questionnaire, the widely used International Physical Activity Questionnaire (IPAQ-SF) seems to have similar or even weaker correlations to accelerometer-measured SB and PA^30,31^. Taken together, questionnaire-based SB and PA estimates cannot directly be compared to accelerometer-measured estimates. However, in the current study the accelerometer data collection lasted for 4 weeks, which may have increased the accuracy of the accelerometer-measured estimates of SB and PA.

The leisure-time MET-index (calculated from additional questions) was not associated with accelerometer-measured SB or PA in this study, but it correlated positively with the Baecke Sport Index. As 45% of the participants reported that they never or seldom play sports during leisure time, the lack of association between the leisure-time MET-index and accelerometry is logical. The leisure-time MET-index questions are focused on physical exercise rather than habitual non-exercise activity and thus, the leisure-time MET-index is not the best estimator of PA or SB in physically inactive individuals.

We additionally tested the effect of gender, i.e. whether men and women estimate their SB or PA differently. There were no gender differences in the Baecke Score or Work, Leisure, and Sport Indexes (Table 1). However, according to accelerometry, men had more sedentary time whereas women spent more time standing compared to men. This has been previously reported and discussed in more detail in our previous articles from the same study sample^21,32^. Regarding individual items of the Baecke questionnaire, there was a gender difference only in one item “*In comparison with others of my age, my physical activity during leisure is (more/less)*”. Thus, it seems that women were more cautious in the estimation of their own PA compared to others, even if they had less accelerometer-measured SB compared to men. It is, however, possible that when answering the questionnaire, they were comparing themselves to other women, not men.

The strength of the current study is the accelerometer data collection period of 4 consecutive weeks with validated hip-worn accelerometers. Compared to the majority of previous similar studies^11,18^, the duration of the data collection period was markedly longer, and thus the risk of random error in the accelerometer measurement was reduced. A weakness is the relatively small sample size, which is, however, partly counteracted with the long accelerometry duration. Another weakness in the current study is the study population, which consisted of physically inactive adults with overweight or obesity, and thus the results can be only applied to a similar population. Moreover, we did not include the information considering occupation in the final Work Index and Baecke Score, as was done in the original questionnaire^10^. However, as it was also discussed in the original publication, this item may be biased by interindividual variation considering different occupations^10^. Thus, we believe that by leaving this items out, we may have increased the validity of the Work Index when compared to accelerometry. Additionally, some of the participants of the current study did not have wage employment, but they considered their daily household or other unpaid work as working time, and therefore they also answered the questions considering working time. This may have strengthened the Work Index compared to the Leisure Index. Finally, due to the large number of correlation analyses, some of the results might be influenced by type 1 error. However, as most correlations were p < 0.01 and the correlations seem plausible in practice, we feel that the results are robust.

## Conclusions

In conclusion, the Baecke Habitual Physical Activity Questionnaire had a statistically significant weak negative correlation with accelerometer-measured SB and significant moderate positive correlations with accelerometer-measured estimates of PA, measured with hip-worn accelerometers during a 4-week period. Thus among inactive adults with overweight or obesity, the questionnaire is useful in estimating daily PA and not as useful in estimating daily SB. Moreover, among self-reportedly inactive adults, Baecke Work and Leisure Indexes are more accurate in the estimation of daily PA compared to the Sport Index. Additionally, the leisure-time MET-index did not significantly correlate with accelerometer-measured SB or PA. This should be considered, when estimating PA with questionnaires in inactive populations. Concentrating solely on leisure-time PA and sports participation may lead to underestimated PA when compared to accelerometry. Furthermore, tiredness after working was associated with less self-reported leisure-time PA; suggesting that different strategies to improve recovery from work-related stress might increase leisure-time PA, or increasing leisure-time PA could reduce tiredness after working. Therefore, work-related stress should probably also be addressed in PA promotion.

## Data Availability

Data are available upon reasonable request from the corresponding author.

## References

[1] Tremblay MS (2017). Sedentary behavior research network (SBRN)—Terminology consensus project process and outcome. Int. J. Behav. Nutr. Phys. Act..

[2] Ekelund U (2020). Joint associations of accelerometer-measured physical activity and sedentary time with all-cause mortality: a harmonised meta-analysis in more than 44 000 middle-aged and older individuals. Br. J. Sports Med..

[3] Dempsey PC (2020). Prospective associations of accelerometer-measured physical activity and sedentary time with incident cardiovascular disease, cancer, and all-cause mortality. Circulation.

[4] Powell C, Herring MP, Dowd KP, Donnelly AE, Carson BP (2018). The cross-sectional associations between objectively measured sedentary time and cardiometabolic health markers in adults—A systematic review with meta-analysis component. Obes. Rev..

[5] Senaratne N, Stubbs B, Werneck AO, Stamatakis E, Hamer M (2021). Device-measured physical activity and sedentary behaviour in relation to mental wellbeing: An analysis of the 1970 British cohort study. Prev. Med..

[6] Hallgren M (2020). Associations of interruptions to leisure-time sedentary behaviour with symptoms of depression and anxiety. Transl. Psychiatry.

[7] Katzmarzyk PT, Friedenreich C, Shiroma EJ, Lee I-M (2022). Physical inactivity and non-communicable disease burden in low-income, middle-income and high-income countries. Br. J. Sports Med..

[8] Prince SA (2020). A comparison of self-reported and device measured sedentary behaviour in adults: a systematic review and meta-analysis. Int. J. Behav. Nutr. Phys. Act..

[9] Skender S (2016). Accelerometry and physical activity questionnaires - a systematic review. BMC Public Health.

[10] Baecke J, Burema J, Frijters J (1982). A short questionnaire for the measurement of habitual physical activity in epidemiological studies. Am. J. Clin. Nutr..

[11] Tebar WR (2022). Validity and reliability of the Baecke questionnaire against accelerometer-measured physical activity in community dwelling adults according to educational level. PLOS ONE.

[12] Mustelin L (2012). Genetic Influences on Physical Activity in Young Adults: A Twin Study. Med. Sci. Sports Exerc..

[13] Aguilar BAS (2022). Leisure-time exercise is associated with lower depressive symptoms in community dwelling adults. Eur. J. Sport Sci..

[14] Kankaanpää A (2021). Leisure-Time and Occupational Physical Activity Associates Differently with Epigenetic Aging. Med. Sci. Sports Exerc..

[15] Kouvonen A (2005). Job strain and leisure-time physical activity in female and male public sector employees. Prev. Med..

[16] Skovgaard EL, Obling K, Maindal HT, Rasmussen C, Overgaard K (2019). Unprompted vigorous physical activity is associated with higher levels of subsequent sedentary behaviour in participants with low cardiorespiratory fitness: a cross-sectional study. Eur. J. Sport Sci..

[17] Helmerhorst HHJ, Brage S, Warren J, Besson H, Ekelund U (2012). A systematic review of reliability and objective criterion-related validity of physical activity questionnaires. Int. J. Behav. Nutr. Phys. Act..

[18] Philippaerts RM, Westerterp KR, Lefevre J (2001). Comparison of Two Questionnaires with a Tri-Axial Accelerometer to Assess Physical Activity Patterns. Int. J. Sports Med..

[19] Philippaerts R, Westerterp K, Lefevre J (1999). Doubly Labeled Water Validation of three Physical Activity Questionnaires. Int. J. Sports Med..

[20] Bull FC (2020). World Health Organization 2020 guidelines on physical activity and sedentary behaviour. Br. J. Sports Med..

[21] Sjöros T (2020). Both sedentary time and physical activity are associated with cardiometabolic health in overweight adults in a 1 month accelerometer measurement. Sci. Rep..

[22] Ainsworth BE (2000). Compendium of Physical Activities: an update of activity codes and MET intensities: *Med*. Sci. Sports Exerc..

[23] Rottensteiner M, Pietiläinen KH, Kaprio J, Kujala UM (2014). Persistence or change in leisure-time physical activity habits and waist gain during early adulthood: A twin-study. Obesity.

[24] Waller K, Kaprio J, Kujala UM (2008). Associations between long-term physical activity, waist circumference and weight gain: a 30-year longitudinal twin study. Int. J. Obes..

[25] Vähä-Ypyä H (2015). Validation of cut-points for evaluating the intensity of physical activity with accelerometry-based mean amplitude deviation (MAD). PLOS ONE.

[26] Vähä-Ypyä H, Vasankari T, Husu P, Suni J, Sievänen H (2015). A universal, accurate intensity-based classification of different physical activities using raw data of accelerometer. Clin. Physiol. Funct. Imaging.

[27] Vähä-Ypyä H, Husu P, Suni J, Vasankari T, Sievänen H (2018). Reliable recognition of lying, sitting, and standing with a hip-worn accelerometer. Scand. J. Med. Sci. Sports.

[28] Stenlund S (2022). Changed health behavior improves subjective well-being and vice versa in a follow-up of 9 years. Health Qual. Life Outcomes.

[29] Mänttäri S (2021). Interventions to promote work ability by increasing physical activity among workers with physically strenuous jobs: A scoping review. Scand. J. Public Health.

[30] Lee PH, Macfarlane DJ, Lam T, Stewart SM (2011). Validity of the international physical activity questionnaire short form (IPAQ-SF): A systematic review. Int. J. Behav. Nutr. Phys. Act..

[31] Craig CL (2003). International physical activity questionnaire: 12-Country reliability and validity: *Med*. Sci. Sports Exerc..

[32] Sjöros T (2021). Influence of the duration and timing of data collection on accelerometer-measured physical activity, sedentary time and associated insulin resistance. Int. J. Environ. Res. Public. Health.

